# A Novel Ab Interno Approach for Traumatic Posterior Globe Perforation: Sutureless Closure With a Single-Layer Human Amniotic Membrane Plug

**DOI:** 10.7759/cureus.97590

**Published:** 2025-11-23

**Authors:** Robert P VanHoy, Samuel D Hobbs, Jefferey C McCloud

**Affiliations:** 1 Medicine, San Antonio Uniformed Services Health Education Consortium, San Antonio, USA; 2 Ophthalmology, Wilford Hall Eye Center, San Antonio, USA; 3 Medicine, Edward Via College of Osteopathic Medicine, Auburn, USA

**Keywords:** human amniotic membrane, intraocular foreign body, open globe injury, proliferative vitreoretinopathy, retinal injury

## Abstract

Intraocular foreign body (IOFB) injuries represent a severe and complex subset of open globe injuries (OGIs). Posterior globe perforations in these injuries present a great surgical challenge for retinal surgeons. Acquiring exposure while taking care not to injure vital structures makes these repairs extremely difficult. There are many ways to approach repair, none of which are straightforward or without consequence. This case report demonstrates a novel, simplified ab interno technique using a single-layer, sutureless human amniotic membrane (hAM) plug to achieve anatomical closure of a large posterior globe perforation following IOFB removal.

## Introduction

Posterior globe perforations, particularly those associated with intraocular foreign bodies (IOFBs), represent one of the most severe and challenging forms of ocular trauma. These injuries are classified as Zone III open globe injuries (OGIs), a category defined by injury extension more than 5 mm posterior to the limbus, and are associated with a significantly poorer visual prognosis compared to more anterior injuries [1,2]. Large ruptures and posterior location lead to poorer outcomes; while smaller perforations can be left to self-heal, larger defects require closure [2]. Without effective closure, the globe cannot maintain intraocular pressure, leading to persistent hypotony and the risk of phthisis bulbi [2]. When performing silicone oil tamponade treatment for posterior perforations, adequate closure is critical for the tamponade to work [3]. Furthermore, prompt and secure wound closure is a critical component of primary OGI repair, which should ideally be performed within 24 hours to minimize the risk of devastating complications such as post-traumatic endophthalmitis [2].

Approaches to closure of posterior perforating injuries in the past have varied. Typical external closure is challenging because the posterior location makes exposure of the defect difficult, and this approach carries a high risk of iatrogenic damage to the macula or optic nerve, especially in a soft, hypotonic eye [2,4]. The inability to safely close a posterior defect externally, coupled with the necessity of a tamponade that will extrude through the unsealed hole, has driven the search for innovative ab interno surgical solutions for posterior perforations. Ab interno techniques aim to seal the perforation from within the vitreous cavity, obviating the need for difficult external dissection. Several ab interno approaches have been described in the literature.

Ab interno suturing

Schmidt et al. described ab interno suturing with 10-0 nylon suture through the scleral wound edges from inside the eye. The technique successfully established integrity with a watertight seal, but was technically demanding and created significant traction when approximating wound edges, potentially leading to distortion of the surrounding tissue [4].

Autologous tissue plugs

Yi et al. reported the successful use of a patient's own Tenon capsule to plug a posterior exit wound. The plugging of the posterior defect eliminated the need for complex suturing, while autologous tissue reduced immunogenicity and leveraged readily available tissue. Initially, the tenon graft’s pliable nature made it difficult to manipulate and secure within the defect, making the initial attempt unsuccessful, requiring a subsequent surgery to place additional packing material for complete closure [5].

Multi-component plugs

Most recently, a case report by Celo et al. describes a novel technique for the internal plugging of a large traumatic posterior perforation. A multi-component internal plug was intravitreally placed, constructed with: donor sclera for structural support, human amniotic membrane (hAM) for biological sealing, and fibrin glue for adhesion. Postoperative testing demonstrated anatomical success of the technique. The multi-component plug remained in place, and there was no migration of silicone oil into the orbit; however, development of proliferative vitreoretinopathy (PVR) led to recurrent retinal detachment, limiting vision recovery [3].

hAMs have a long history of use in ocular surface reconstruction, while their application in vitreoretinal surgery is a more recent but rapidly evolving field. The amniotic membrane is prized for its low immunogenicity, allowing it to be used as an allograft without inducing a significant rejection response. It also exhibits potent anti-inflammatory, anti-fibrotic, and anti-angiogenic properties, which are highly desirable in the post-traumatic ocular environment [6]. Intravitreally, hAMs have been used to help close macular holes, retinal detachments, and large macular tears [7,8]. Caporossi et al. studied eyes with recurrent macular holes and eyes with retinal detachments; both patient groups showed significant vision improvement. Caporossi demonstrated that optical coherence tomography (OCT) in the patients showed neuroretinal ingrowth over the amniotic membrane plug [7]. In vitro studies have further demonstrated that retinal pigment epithelium (RPE) cells can seed over an amniotic plug in as little as 24 hours. The cell growth also showed epithelial phenotype morphology and upregulation of important growth factors that are critical in maintaining retinal homeostasis [9].

## Case presentation

Initial presentation and diagnosis

A 35-year-old male presented to the emergency room on transfer for a left IOFB and open globe. The injury was sustained while using an angle grinder without eye protection, at which time he felt an object strike his left eye, followed by an immediate and profound loss of vision.

Visual acuity (VA) was 20/20 on the right and counting fingers at 1 ft on the left. Intraocular pressure was 16 mmHg on the right and deferred on the left. Slit-lamp examination demonstrated a horizontal 4-mm, full-thickness corneal laceration at 3 o’clock with a peaked iris, frank hyphema, and a traumatic cataract with lens material in the anterior chamber on the left. 

**Figure 1:**
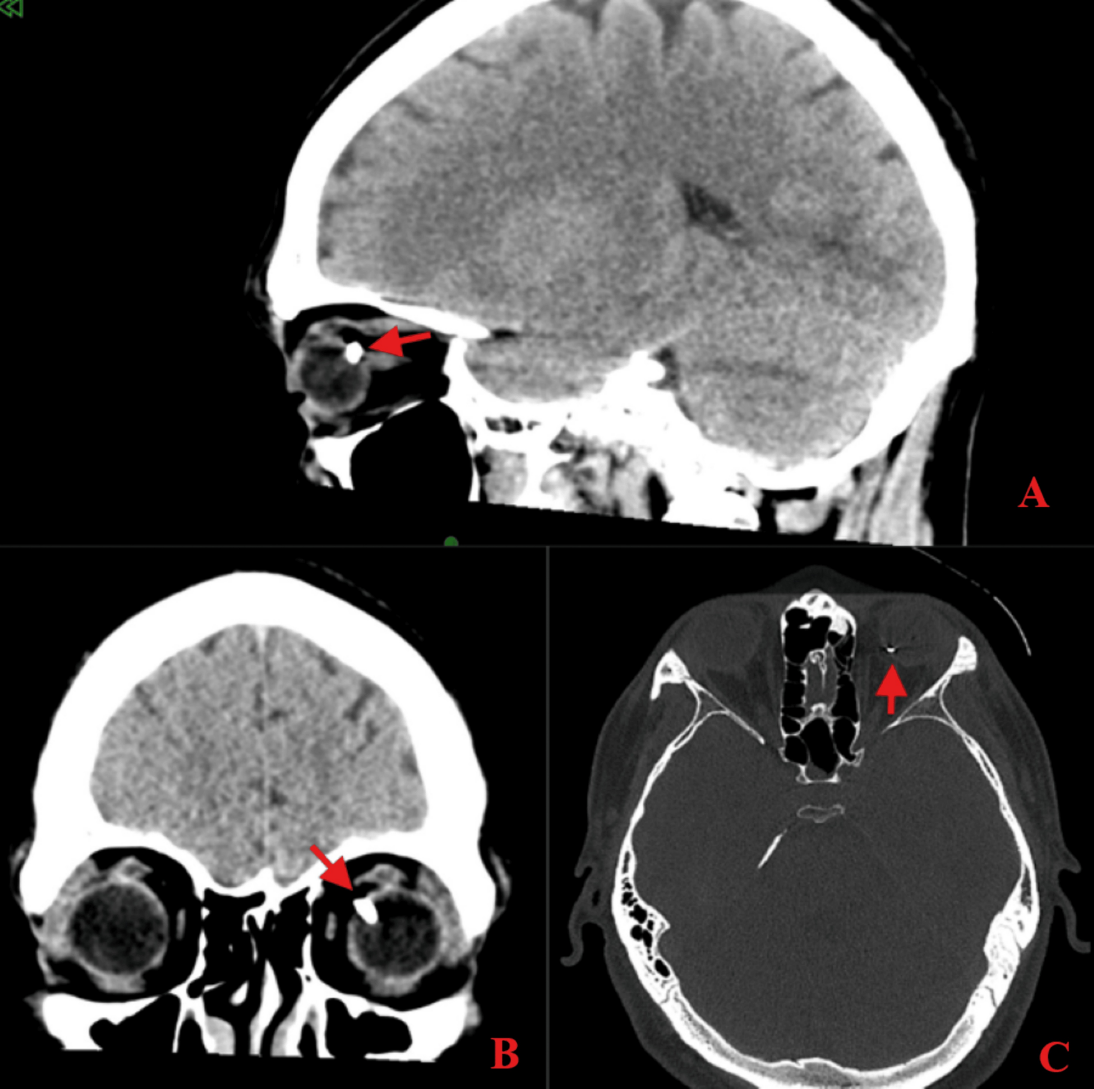
Preoperative computed tomography Sagittal (A), coronal (B), and axial (C) CT views of the face and orbits displaying left open globe, injury. Red arrow - a radiopaque foreign body was visualized within the posterior superior aspect of the left globe, including evidence of retinal penetration, and a small foci of free air within the episcleral space.

A computed tomography (CT) scan of the face and orbits showed a left OGI with rupture and silocation of the left lens. Within the posterior superior aspect of the globe, there was a radiopaque foreign body that penetrated the retina superior to the optic nerve. Small foci of free air were noted within the episcleral space, consistent with a full-thickness perforation (Figure 1). The patient was admitted to the hospital and consented to exploration and repair in the morning.

Operation 1: primary repair, IOFB removal, and hAM plug placement

IOFB Removal

A 360-degree exam using the resight system was performed to reveal multiple retinal breaks in the periphery, as well as a large metallic 6.5 × 2 × 1.5 mm IOFB protruding through the retina and sclera superior to the optic nerve. The IOFB was carefully disembedded and removed through the clear corneal incision using a rare-earth magnet and IOFB forceps. 

Primary Repair

Anterior repair of the 4-mm corneal laceration was closed with five 10-0 nylon simple interrupted corneal sutures placed to seal the corneal laceration. There was no apparent leak, and the anterior chamber was well-formed. The anterior capsule was noted to be violated nasally, so capsulorrhexis forceps and a cystotome were used to extend the ruptured flap 360 degrees to form a capsulotomy. The irrigation and aspiration device was then used to remove the bulk of the lens nucleus and cortex, followed by pars plana lensectomy to ensure full removal of the lens. Posterior exploration revealed multiple horseshoe tears in all quadrants and a posterior penetration site superior to the optic nerve. Two inferior peripheral iridotomies were created with the cutter inferiorly to prevent reverse pupillary block caused by the high pressure from the tamponade agent in the now aphakic patient after lensectomy. A core vitrectomy, followed by pars plana vitrectomy, was performed. Endolaser photocoagulation was applied to create a barricade around all identified peripheral retinal breaks.

hAM Plug Placement

**Figure 2:**
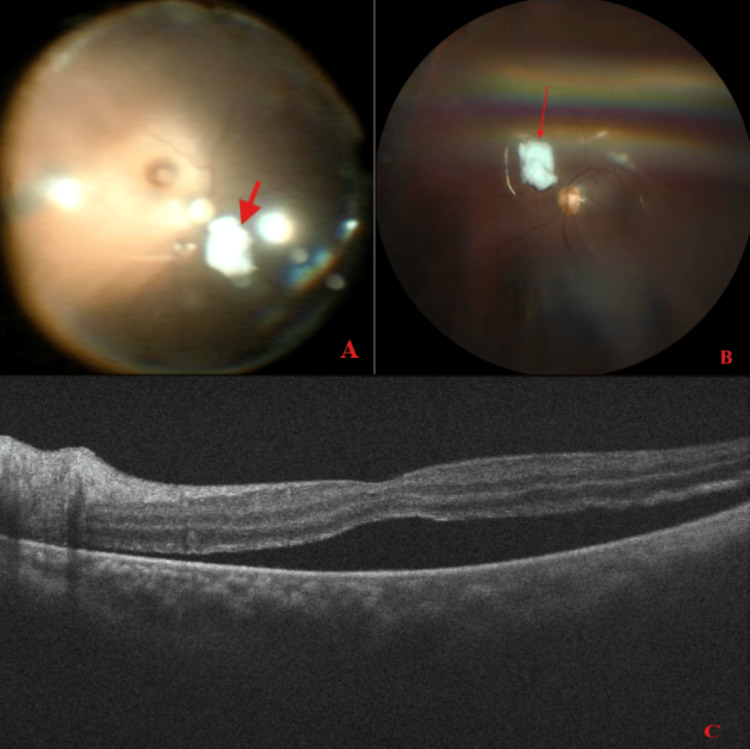
Intraoperative and postoperative fundus imaging (A) Intraoperative fundus view of the left eye showing a human amniotic membrane (red arrow) graft over the posterior scleral laceration in the inferior view of the optic nerve. (B) Post-second operation day one fundus photo of the left eye showing human amniotic membrane (red arrow) superior and adjacent to the optic nerve. (C) Post-second operation week two optical coherence tomography of the left eye showing subretinal fluid, demonstrating macula-off detachment.

When performing the intravitreal fluid infusion, it was noted that the fluid was exiting the posterior scleral laceration and causing significant eye collapse, proptosis, and orbital compartment syndrome. Air-fluid exchange and a conjunctival cutdown were performed to relieve intraorbital pressure. The proptosis improved, as did the eye contour. Knowing that the globe would not support an intraocular gas or silicone oil tamponade, the decision was made to intravitreally place an amniotic membrane graft over the posterior scleral laceration. A hAM graft was prepared, and a 1.5 × 1cm piece was cut. Visualization of the defect was extremely challenging, so the decision was made not to further trim the graft at the time to broadly cover the suspect area. Using Maxgrip forceps, the graft was then placed over the suspect area. Beginning an air-gas exchange with 16% C3F8 gas, the globe started to take shape, indicating that the graft was in the correct position (Figure 2A). Throughout gas-air exchange, great attention was given to monitoring for globe leak and to keep the soft tip aspiration canula away from the graft. On postoperative day one, the hAM could be visualized, and the globe was successfully closed and holding pressure. This demonstrated that the surface tension from the tamponade agent was sufficient to hold the hAM graft plug in place.

Operation 2: retinal detachment and repair

Retinal Detachment

Postoperative week 2, the patient was found to have a rhegmatogenous retinal detachment involving the macula that appeared to be related to insufficient positioning post-operatively, with no significant PVR. The patient then consented to the repair and placement of an intraocular lens. 

Repair

A repeat pars plana vitrectomy was performed, and a three-piece intraocular lens was placed in the ciliary sulcus. Air-oil exchange with 1000 centistoke silicone oil was then performed to provide long-term tamponade (Figure 2B). On the first day of follow-up, the retina was flat and attached under silicone oil.

Operation 3: PVR and definitive management

PVR

Two weeks later, it was discovered that the patient had a retinal break and macula-involving retinal detachment, this time caused by PVR inferiorly (Figure 2C). The patient consented to retinectomy and was taken for surgery.

Definitive Management

Silicone oil was removed using a side-port blade and the soft-tip cannula. Inferior 180-degree retinectomy was performed, and hemostasis was achieved with diathermy. Perfluorocarbon liquid was placed, and the retina was noted to flatten well without any evidence of residual traction. Endolaser was used to barricade the entire retinectomy edge with confluent rows of laser. The eye was refilled with 1000 centistoke silicone oil. As an adjunctive therapy to suppress further cellular proliferation, an intravitreal injection of methotrexate (400 μg in 0.1 mL) was administered at the end of the surgery. 

Final outcome

At the most recent follow-up visit, the patient's retina remained attached 360 degrees under silicone oil tamponade. The hAM plug was stable in its position, and the globe was anatomically intact with normal intraocular pressure. The patient's final best-corrected VA was improved to 20/100 under oil.

## Discussion

Novel ab interno techniques such as ab interno suturing, three-layer plugs, and Tenon plugs have been reported with some success, while each has its own difficulties [3-5]. This case report demonstrates sufficient closure of a traumatic IOFB with posterior globe perforation by a single-layered hAM plug alone. By sealing the defect, the plug allowed for the completion of the vitrectomy and the successful installation of a gas tamponade, which would have otherwise been impossible. This demonstrates a significant simplification of the internal plugging concept, offering an effective solution without the need for sutures, multiple biomaterials, or complex intraocular assembly. The stability of the plug was confirmed throughout the patient's subsequent two surgeries, underscoring its durability as a structural seal. Later development of PVR with retinal detachment demonstrates the need to mitigate the common maladaptive biological response in OGIs [10]. Commonly, PVR in trauma affects the location of the defect, but interestingly, PVR did not develop near the hAM graft, possibly outlining hAM's anti-inflammatory and anti-fibrotic properties [11]. This case introduces a new, less complex option to the vitreoretinal surgeon's armamentarium for managing these challenging injuries. The technique's relative simplicity becomes apparent when compared to previously described ab interno methods (Table 1) [3-5].

**Table 1:** Comparison of ab interno techniques for posterior globe perforation closure Comparing ab interno techniques to repairing posterior globe perforations [3-5].

Feature	Ab Interno Suturing	Autologous Tenon Capsule	Multi-Layer Plug	Single-Layer hAM Plug (Present Case)
Technical Complexity	High (bimanual intravitreal suturing)	Moderate (autologous tissue harvesting and placement)	High (intraocular assembly of multiple components)	Low (single component placement)
Materials	10-0 Nylon Suture	Autologous Tenon's Capsule	Donor Sclera, hAM, Fibrin Glue	Human Amniotic Membrane (hAM)
Tissue Distortion	Potential for traction and tissue distortion	Minimal	Minimal (packing)	Minimal
Reported Outcome	Anatomical closure, limited VA	Anatomical closure, normal IOP	Anatomical closure, limited VA (PVR)	Anatomical closure, limited VA (PVR)
Key Advantage	Strong mechanical closure	No immunogenicity	Combined structural and biological support	Simplicity, material availability, biological properties

Future work could explore impregnating hAM grafts with sustained-release anti-fibrotic or anti-inflammatory medications. This would transform the graft from a passive scaffold into an active therapeutic delivery platform, designed to temper the eye's aggressive healing response that leads to PVR [12]. The most promising trait of hAM, however, is its regenerative potential [6]. Further research should find ways to integrate hAM as a carrier matrix for regenerative elements, such as retinal progenitor cells or stem cells.

## Conclusions

Achieving anatomical stability when treating large traumatic posterior globe perforation injuries is necessary, but it does not guarantee functional success. This case demonstrates the use of readily available hAMs alone to be an effective and relatively simple way to treat these difficult cases. Their unique structure also offers a framework for the recovery of retinal cells. Its use, like in many ocular trauma cases, is limited by recurrent detachment from PVR. However, the simplicity makes the technique more accessible and reproducible, offering a practical solution for a difficult surgical problem. Continued research on similar cases may offer a new path of care when approaching such serious and difficult ocular trauma.
