# Correction: A Novel Ab Interno Approach for Traumatic Posterior Globe Perforation: Sutureless Closure With a Single-Layer Human Amniotic Membrane Plug

**DOI:** 10.7759/cureus.c388

**Published:** 2025-12-23

**Authors:** Robert P VanHoy, Samuel D Hobbs, Jefferey C McCloud

**Affiliations:** 1 Medicine, San Antonio Uniformed Services Health Education Consortium, San Antonio, USA; 2 Ophthalmology, Wilford Hall Eye Center, San Antonio, USA; 3 Medicine, Edward Via College of Osteopathic Medicine, Auburn, USA

This article has been corrected to include Samuel D. Hobbs as an author.

